# Endogenous asymmetric dimethylarginine accumulation contributes to the suppression of myocardial mitochondrial biogenesis in type 2 diabetic rats

**DOI:** 10.1186/s12986-020-00486-4

**Published:** 2020-08-24

**Authors:** Yan Xiong, Chun-Xia Hai, Wei-Jin Fang, Yan-Ping Lei, Xiao-Mei Li, Xin-Ke Zhou

**Affiliations:** 1 Department of Central Laboratory, The Fifth Affiliated Hospital of Guangzhou Medical University, Guangzhou, 510700 Guangdong China; 2 Guangzhou Institute of Snake Venom Research, Guangzhou Medical University, Guangzhou, 511436 Guangdong China; 3 Department of Pharmacology, School of Pharmaceutical Sciences, Central South University, Changsha, 410078 Hunan China

**Keywords:** Asymmetric dimethylarginine, Diabetic cardiomyopathy, Mitochondrial biogenesis, Peroxisome proliferator-activated receptor-γ coactivator-1α

## Abstract

**Background:**

Suppressed mitochondrial biosynthesis has been reported to be the early signal of mitochondrial dysfunction which contributes to diabetic cardiomyopathy, but the mechanism of mitochondrial biosynthesis suppression is unclear. Nitric oxide synthase inhibitor asymmetric dimethylarginine (ADMA) is closely related to diabetic cardiovascular complications. This study was to determine whether endogenous ADMA accumulation was involved in the suppression of myocardial mitochondrial biogenesis in diabetic rats and to elucidate the potential mechanism in rat cardiomyocytes.

**Methods:**

Type 2 diabetic rat model was induced by high-fat feeding plus single intraperitoneal injection of small dose streptozotocin (35 mg/kg). The copy number ratio of mitochondrial gene to nuclear gene was measured to reflect mitochondrial biogenesis. The promoter activity of peroxisome proliferator-activated receptor-γ coactivator-1α (PGC-1α) and its post-translational modifications were detected by dual-luciferase reporter assay and immunoprecipitation.

**Results:**

Myocardial ADMA content was enhanced and associated with suppressions of myocardial mitochondrial biogenesis and cardiac function in parallel with PGC-1α downregulation and uncoupling protein 2 (UCP2) upregulation in the myocardium of diabetic rats compared with control rats. Similarly, ADMA and its homolog could inhibit myocardial mitochondrial biogenesis and PGC-1α expression, increase UCP2 expression and oxidative stress in vitro and in vivo. Moreover, ADMA also suppressed the promoter activity and PGC-1α expression but boosting its protein acetylation and phosphorylation in rat cardiomyocytes.

**Conclusions:**

These results indicate that endogenous ADMA accumulation contributes to suppression of myocardial mitochondrial biogenesis in type 2 diabetic rats. The underlying mechanisms may be associated with reducing PGC-1α promoter activity and expression but boosting its protein acetylation and phosphorylation.

## Background

Diabetes mellitus is a major global health problem, especially in newly industrialized and fast developing nations such as China [1]. Diabetic patients are predisposed to cardiovascular diseases, especially diabetic cardiomyopathy (DCM), which has become the principal cause of diabetes-related morbidity and mortality. But the mechanisms underlying DCM were remained obscure. Emerging evidence suggests that mitochondrial dysfunction plays a crucial role in the pathogenesis of DCM [2, 3].

Mitochondria are known as the powerhouses of eukaryotic cells. The most prominent function of mitochondria is to produce energy in the form of ATP through oxidation-phosphorylation, which can be destroyed by the uncoupling protein 2 (UCP2). As a proton channel protein located in mitochondrial inner membrane, UCP2 can mediate protons leak leading to the uncoupling of oxidation phosphorylation and the reduction of mitochondrial ATP (mtATP) production [4, 5]. Therefore, UCP2 plays an important role in the dysfunction of mitochondrial energy metabolism. On other hand, UCP2 has a favorable effect on reducing mitochondrial reactive oxygen species (ROS) generation by uncoupling of oxidation-phosphorylation. Consequently, mitochondrial dysfunction is associated with not only the decrease of ATP production but also the increase of ROS generation. Furthermore, mitochondrial dysfunction is closely related to the suppression of mitochondrial biogenesis. It has been documented that peroxisome proliferator-activated receptor-γ coactivator-1α (PGC-1α) is a master regulator of mitochondrial biogenesis through co-activating the transcription of peroxisome proliferator-activated receptor-gamma (PPARγ) for promoting and maintaining replication and transcription of mitochondrial DNA (mtDNA) [6, 7]. PGC-1α is also subject to extensive post-translational modifications such as acetylation, phosphorylation and so on. The acetylation of PGC-1α is predominantly catalyzed by acetyl transferase resulting in a transcriptionally inactive protein, and deacetylation of PGC-1α is accomplished by silent information regulator 2 homolog 1 (Sirt1) leading to an increase of its transcriptional activity [8]. The phosphorylation of PGC-1α is achieved by many kinases such as adenosine monophosphate-activated protein kinase (AMPK) and protein kinase B (Akt). The former-catalyzed phosphorylation of PGC-1α may lead to a more stable and active protein, whereas the latter result in the decrease of its stability and transcriptional activity [9, 10]. Moreover, mitochondrial biogenesis is also regulated by nitric oxide (NO) derived from endothelial nitric oxide synthase (eNOS). NO not only mediated the up-regulation of PGC-1α expression and mitochondrial biogenesis in brown adipose tissue stimulated by cold exposure [11], but also participated in calorie restriction-induced increases of mitochondrial DNA content, PGC-1α expression and ATP production in white adipose tissue and other tissue [12]. NO facilitated these effects through interaction with Sirt1 and AMPK in skeletal muscles [12, 13]. NO donor could directly upregulate PGC-1α expression and mitochondrial biogenesis in cultured adipocytes and myotubes [11, 13]. Accordingly, NO is an effector molecule for up-regulation of PGC-1α and mitochondrial biosynthesis in response to diverse stimulations. Several studies have demonstrated impaired mitochondrial biosynthesis and function in the myocardium of type 1 and type 2 diabetic animals [2, 3]. Even the suppressed mitochondrial biogenesis and impaired mitochondrial activity have been reported to occur in skeletal muscle of young, lean, normoglycemic, insulin-resistant offspring of parents with type 2 diabetes mellitus (T2DM) [14, 15]. Consequently, decreased mitochondrial biogenesis has been recognized as not only an initial factor of mitochondrial dysfunction in diabetes but also a hallmark of high cardiovascular risk in metabolic diseases [16, 17].

However, the reason why mitochondrial biogenesis is suppressed in diabetic myocardium is unclear. An increasing number of studies have indicated that endogenous asymmetric dimethylarginine (ADMA) accumulation is associated with cardiovascular diseases and diabetic cardiovascular complications [18–20]. ADMA as an endogenous competitive inhibitor of nitric oxide synthase (NOS) not only reduces NO production but also increases superoxide generation by uncoupling NOS. After extensive prevalent cohort, prospective and cross-sectional studies, ADMA has been identified as a marker of cardiovascular risk. Subsequently, ADMA has also been reported to be involved in the mitochondrial dysfunction in ovine fetal pulmonary artery endothelial cells and in the liver of diabetic rats [21, 22]. However, most of above studies are limited to the correlation between elevated serum ADMA concentrations and diabetic cardiovascular complications. It is less known the direct role of ADMA in the heart or myocardial tissue, and it is even not clear how ADMA causes diabetic cardiovascular complications. Therefore, this study was sought to reveal the important effect of ADMA on mitochondrial biogenesis and function in the myocardium of diabetic rats and to explore the potential mechanisms underlying ADMA-induced impairments of mitochondrial biogenesis and function in rat cardiomyocytes, all of which will help to elucidate the pathogenesis of DCM. In addition, rosiglitazone as an agonist of PPARγ has been reported to improve mitochondrial biogenesis and PGC-1α expression in the adipose tissue of spontaneous genetic diabetic db/db mice and high-fat diet–fed mice [23]. Hence this study was also to determine whether rosiglitazone could reverse ADMA-induced suppression of mitochondrial biogenesis and function in cardiomyocytes in order to confirm ADMA-induced inhibition of mitochondrial biogenesis via PGC-1α/PPARγ pathway. Overall speaking, this study would provide a novel insight into the pathogenesis of DCM, the potential mechanism underlying the suppression of myocardial mitochondrial biogenesis in diabetes, and the future development of new strategies for clinical diagnosis, prevention and treatment of DCM.

## Materials and methods

### Animal experiments

All animal experiments were approved by the Experimental Animal Administration Committee of Guangzhou Medical University and carried out in accordance with the National Institutes of Health guidelines. Specific pathogen-free male Sprague-Dawley rats weighing 180 ~ 200 g at 2 months of age were purchased from Guangdong Medical Laboratory Animal Center (Guangzhou, China).

#### Rat model of T2DM

After 1w adaptive feeding with regular chow, rats were randomly assigned to commercially available regular chow (Control) and in-house prepared high-fat diet (HFD, 60% chow, 10% lard, 10% egg yolk powder, 1.5% cholesterol and 0.1% sodium cholate). Rat model of T2DM was induced by intraperitoneal injection of a low dose STZ (35 mg/kg dissolved in 0.1 mol/L citrate buffer at pH 4.5) after feeding HFD for 3w as previously described [24], while control rats were given an equal volume of citrate buffer. The onset of diabetes was confirmed by the presence of glycosuria and the following two consecutive measurement of blood glucose concentration (≥16 mmol/L) in randomly on every other day. Diabetic rats were kept on HFD for another 10w while control rats continued regular chow for the same period.

#### Oral glucose tolerance test

By the end of the experiment, oral glucose tolerance test (OGTT) was performed as previously indicated [25]. The fasting blood glucose (FBG) and additional blood samples were collected at 30, 60, 90 and 120 min after glucose loading (2.0 g/kg). The area under the curve (AUC) of blood glucose concentration was calculated by means of the proximity ladder shaped formula. Fasting plasma insulin (FIns) concentration was measured by ELISA kit, and insulin sensitivity index (ISI) was also calculated with FBG and FIns (ISI = 1/FBG × FIns). Serum lipid profiles including triglyceride (TG), total cholesterol (TC), low density lipoprotein-cholesterol (LDL-C) and high density lipoprotein-cholesterol (HDL-C) concentrations were assayed by spectrophotometric methods with respective commercial kits (All commercial kits of biochemical examination from Beyotime Biotechnology, Shanghai, China).

#### Rat model of exogenous NOS inhibitor

Rats were randomly divided into control and model groups after 1w adaptive feeding. The model rats were administrated by gavage of exogenous NOS inhibitor L-nitro-arginine (L-NNA, 50 mg/kg/d), a homolog of ADMA for 8w, while control rats were given an equal volume of physiological saline for the same period. All rats were caged with regular chow and water ad libitum under standard laboratory conditions with a 12 h light/dark cycle.

### Echocardiography

Changes in cardiac geometry and function of T2DM rats (at about 6.5 months of age) were evaluated at the end of experiments using echocardiography (Vevo® 2100 system, Visual Sonics Inc., Toronto, Canada) equipped with a high-frequency (15-50 MHz) Micro Scan array transducer (Visual Sonics MS-250, Visual Sonics Inc., Toronto, Canada) as described by Ramirez E et al [26]. Rats were anesthetized by intraperitoneal injection of chloral hydrate (400 mg/kg), and transthoracic M-mode examinations at the papillary muscle level were performed to determine left ventricular systolic function along with morphology and structure. Doppler echo was applied to measure left ventricular diastolic function of rats. After finishing the echocardiography, blood (8 ~ 10 ml/rat) was drawn from carotid artery intubation to separate plasma or serum for biochemical measurements, and then hearts were quickly excised for western blotting and PCR analyses. Heart histology was also performed in 2 ~ 3 mm sections stained with hematoxylin and eosin and photographed.

### Cells culture

The H9C2 embryonal rat heart-derived cell line was obtained from the Institute of Biochemistry and Cell Biology, the Chinese Academy of Sciences (Shanghai, China) and cultured in DMEM supplemented with 10% FBS under 37 °C in a humidified atmosphere of 5% CO2 as previously described [26]. The third passage cells from cryopreservation began to seed into 6-well plates for gene transcription and dual-luciferase reporter assay.

Primary cardiomyocytes were isolated from hearts of 2 ~ 3 days neonatal Sprague-Dawley rats obtained from the Guangdong Medical Laboratory Animal Center. After rats were anesthetized with 3% isoflurane mixed with oxygen in a chamber, hearts were aseptically removed, cut into 1mm^3^ pieces and repeatedly digested with 0.1% type II collagenase (Worthington, USA) under 37 °C. Digest solutions were collected, centrifuged and resuspended in DMEM containing 10% FBS and incubated in 5% CO_2_ incubator under 37 °C for 40 ~ 60 min to permit removal of the most of the rapidly adhering fibroblasts. The supernatants were planted into 6-well plates at the density of 5 × 10^5^ cells/ml and cultured with DMEM containing 10% FBS and 100 nmol/L 5′-bromide uracil (Brdu), which suppressed the growth of cardiac fibroblasts, in 5% CO_2_ incubator under 37 °C for measurements of protein expression and protein post-translational modification.

After growing to 70% confluency, cells were incubated with various dose of ADMA (3 ~ 100 μM) for different time (12 ~ 72 h) in experiments of dose-response and time course. In drug-treated groups, cells were pretreated with drugs for 2 h and then co-incubated with ADMA or high glucose for another 48 h, respectively. After finishing these treatments, cells were harvested for the measurements of gene transcription, protein expression and protein post-translational modification.

### Mitochondrial assay

Mitochondria isolation from rat hearts or cardiomyocytes was performed according to the methods as previously reported [16, 21]. In briefly, myocardial tissue (30 mg) from the apical of fresh harvested rat heart was minced and homogenized by a motor-driven homogenizer in the isolation buffer containing 250 mM sucrose, 0.1 mM KCL, 1 mM Na_2_EDTA, and 0.1 mM Tris-HCl with pH 7.4. The homogenate was centrifuged at 800×g for 10 min under 4 °C, and the supernatant was centrifuged again at 10,000×g for 10 min under 4 °C. The pellet was washed with isolation buffer and gently resuspended in buffer (30 μl) containing 0.1 mM KCL and 0.1 mM Tris-HCl for mitochondrial assays. Cultured cardiomyocytes in each well of 6-well plates were respectively homogenized mechanically after digestion with trypsin and suspended in 0.5 ml ice-cold hypotonic buffer containing 10 mM HEPES, 10 mM MgCl_2_ and 42 mM KCl. The supernatant was centrifuged at 800×g for 5 min under 4 °C, and the resultant supernatant was centrifuged again at 15,000×g for 15 min under 4 °C. The mitochondrial pellet was resuspended in buffer (30 μl) containing 0.25 mM sucrose and 10 mM Tris with pH 7.0 for mitochondrial assays.

Mitochondrial biogenesis was evaluated by mtDNA content, which was mirrored by the copy number ratio of mitochondrial genes like cytochrome C oxidase subunit I (COX I) to the nuclear genes such as β-actin indirectly [21]. Briefly, the total genomic DNA of heart tissue or cells was extracted with proteinase K digestion followed by phenol-chloroform extraction. PCR amplification was carried out using the extracted total genomic DNA as templates and specific primers of COX I and β-actin genes (Table 1) at the reaction conditions of PCR (Table 2).

**Table 1:** PCR primer sequences

Genes	Primer sequence	PCR products
COX I	Sense: 5′-GCCTAGATGTAGACACCCGAGCC-3′ Anti-sense: 5′-CGACG AGGTATCCCTGCTAATCC-3’	430 bp
β-actin	Sense: 5’-TAAAGACCTCTATGCCAACACAGT-3′ Anti-sense: 5′-CACGATGGAGGGGCCGGACTCATC-3’	241 bp
PGC-1α	Sense: 5’-AAGGTCCCCAGGCAGTAGAT-3′ Anti-sense: 5′-GCGGTCTCTCAGTTCTGTCC-3’	325 bp
UCP2	Sense: 5’-GAGCACTGTCGAAGCCTACA-3′ Anti-sense: 5′-GAGGTCGTCTGTCATGAGGT-3’	367 bp
GAPDH	Sense: 5’-CTACCCACGGCAAGTTCAAC-3′ Anti-sense: 5′-CCAGTAGACTCCACGACATAC-3’	622 bp

**Table 2:** PCR reaction conditions

Genes	Initial denature	Cycle parameters	Final extension	Cycle number
COXI	94 °C 5 min	94 °C 30 s; 60 °C 30 s; 72 °C 45 s	72 °C 10 min	25
PGC-1α	94 °C 5 min	94 °C 30s; 64 °C 45 s; 72 °C 45 s	72 °C 10 min	30
UCP2	94 °C 5 min	94 °C 30s; 55 °C 30 s; 72 °C 1 min	72 °C 10 min	30

The content of mtATP was measured to reflect mitochondrial function according to the manufacturer’s instructions of ATP-dependent bioluminescence assay kit. The mixture of 30 μl of mitochondrial extract solution and 300 μl of lysis buffer was centrifuged at 12000×g for 10 min under 4 °C.The resulting supernatant (100 μl) was used for the analysis of ATP content, which was normalized to the protein content of the mitochondrial extract solution [21].

### RT-PCR and luciferase assay

Reversed transcription polymerase chain reaction (RT-PCR) was carried out with specific primers of PGC-1α and UCP2 genes (Table 1) at the reaction condition of PCR (Table 2). The final PCR products were separated on a 1% agarose gel and visualized by ethidium bromide staining. The optical densities of mRNA bands were scanned and normalized to GAPDH as an internal control as previously reported [20].

Dual-luciferase reporter assay was employed to test the activity of PGC-1α promoter as previously described [27]. In brief, PGL4-PGC-1α promoter luciferase plasmid (encoding wild type PGC-1α promoter sequences upstream of luciferase reporter gene) or PGL negative control plasmid and internal control Renilla-luciferase plasmid (pRL-TK) were transfected into H9C2 cells with lipofectamin™ 2000. And 6 h later, cells were incubated with high glucose (30 mM) or ADMA (30 μM) for 48 h, respectively. Cells were lysed, and 20 μl cell lysates were used for luciferase measurement, which was performed according to the instructions of Dual-Luciferase Reporter Assay kit (Genecopoeia, Rockville, MD, USA). The final data were normalized by dividing firefly luciferase activity with that of Renilla-luciferase.

### Western blotting and immunoprecipitation

Myocardial tissue and cardiomyocytes were lysed with RIPA buffer for the determination of protein arginine N-methyltransferase 1 (PRMT1), dimethylarginine dimethylaminohydrolase1 (DDAH1) or DDAH2, eNOS, PGC-1α, UCP2 and β-actin (the former three antibodies were products of Abcam, Cambridge, MA, USA from rabbit, goat, goat, respectively; and the latter four antibodies from Santa Cruz Biotechnology, Santa Cruz, CA, USA from rabbit, rabbit, goat, rabbit, respectively) expression by Western blotting as described previously [25].

Immunoprecipitation was employed to detect post-translational acetylation and phosphorylation of PGC-1α as described by Lei S et al [28]. In this experiment, a total of 400 μl cell lysates were subjected to immunoprecipitation with 1 μg PGC-1α primary antibody at 4 °C overnight. The antibody-bound proteins were precipitated with 15 μl protein G magnetic beads. After separating from protein G magnetic beads, immunoprecipitates were subjected to Western blotting as described above with the antibody against acetylated-lysine or phosphorylated-serine/threonine (protein G magnetic beads and antibodies were products of Cell signaling technology, Danvers, MA, USA, the latter from rabbit), respectively.

### Biochemical examination

Serum ADMA concentration was measured by high-performance liquid chromatography, and myocardial ADMA content was detected by ELISA method. Myocardial activities of DDAH and NOS as well as contents of nitric/nitrate were measured in tissue and cells using their commercial kits as previous described [20]. The activity of manganese-superoxide dismutase (Mn-SOD), an antioxidant enzyme located in mitochondria, and the content of malondialdehyde (MDA), derived from lipid peroxidation were examined with their commercial kits to evaluate oxidative stress, protein contents in the supernatants of tissue homogenates and cell lysis were measured by Bicinchoninic acid Protein Assay with the commercial kit and used to normalize activities of DDAH, NOS and Mn-SOD as well as contents of nitric/nitrate and MDA as previously described [25].

### Statistical analysis

Data are expressed as mean ± *S.E.M.*, and the statistical analysis was performed using one-way ANOVA followed by the Newman-Keuls test. Linear regression was conducted to evaluate the possible correlation between cardiac ADMA contents and cardiac function indexes or mitochondrial function parameters. Pearson’s correlation coefficients were calculated, and *P* < 0.05 was considered as statistical significance.

## Results

### Identification of T2DM rat model

**Fig. 1:**
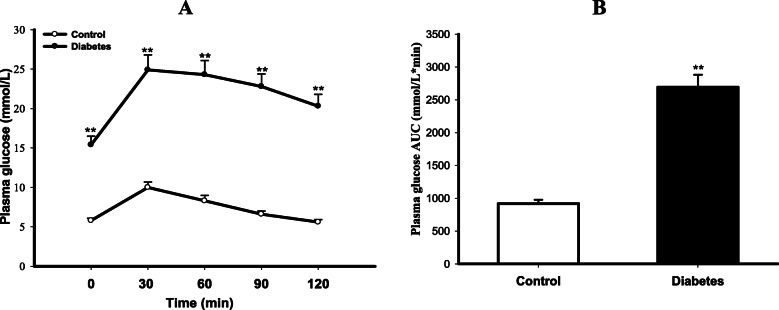
Impairment of glucose tolerance in T2DM rats. The glucose tolerance of control and type 2 diabetes mellitus (T2DM) rats was evaluated by oral glucose tolerance test (OGTT), and the area under concentration-time curve (AUC) of blood glucose concentration was calculated by the proximity ladder shaped formula: AUC (mmol/L*min) = 1/2 × (BG 0 min + BG 30 min) × 30 min + 1/2 × (BG 30 min + BG 60 min) × 30 min + 1/2 × (BG 60 min + BG 90 min) × 30 min + 1/2 × (BG 90 min + BG 120 min) × 30 min. A is the plasma glucose levels, and B is the AUC. Data are expressed as mean ± *S.E.M.*, *n* = 5. **P < 0.01 vs Control

As shown in Fig. 1, the blood glucose level at every time point of OGTT was much higher in diabetic rats than that in control rats (*P* < 0.01). Calculated AUC of blood glucose concentration was also larger in diabetic group (*P* < 0.01 vs Control), indicating the impairment of glucose tolerance in diabetic rats. As displayed in Table 3, diabetic rats showed not only significant elevations in FBG and FIns levels, but also abnormal lipid metabolism in terms of a lower HDL-C and higher TG, TC and LDL-C concentrations in serum than those in control rats (All *P* < 0.01). The ISI calculated by FBG and FIns was remarkably decreased, indicating insulin resistant in diabetic rats. Taken together, these results demonstrated the successful establishment of T2DM rat model.

**Table 3:** Changes in blood glucose, plasma insulin, ISI and serum lipid profiles in T2DM rats

Groups	FBG (mmol/L)	FIns (mU/L)	ISI (×10^−3^)	TG (mmol/L)	TC (mmol/L)	LDL-C (mmol/L)	HDL-C (mmol/L)
**Control**	7.00 ± 0.28	33.17 ± 4.01	4.80 ± 0.69	0.60 ± 0.02	1.25 ± 0.17	0.67 ± 0.11	0.47 ± 0.03
**T2DM**	20.74 ± 2.32^**^	68.73 ± 5.33^**^	0.83 ± 0.14^**^	2.98 ± 0.19^**^	3.13 ± 0.43^**^	2.64 ± 0.16^**^	0.34 ± 0.02^**^

Levels of fasting blood glucose (FBG), fasting plasma insulin (FIns) and lipid profiles in serum including triglyceride (TG), total cholesterol (TC), low density lipoprotein-cholesterol (LDL-C) and high density lipoprotein-cholesterol (HDL-C) were assayed spectrophotometrically with respective commercial kits in control and type 2 diabetic (T2DM) rats. Insulin sensitivity index (ISI) was calculated with FBG and FIns (ISI = 1/FBG × FIns). Data are expressed as mean ± *S.E.M.*; *n* = 5. **P < 0.01 vs Control

### Impairment of cardiac function in T2DM rats

**Fig. 2:**
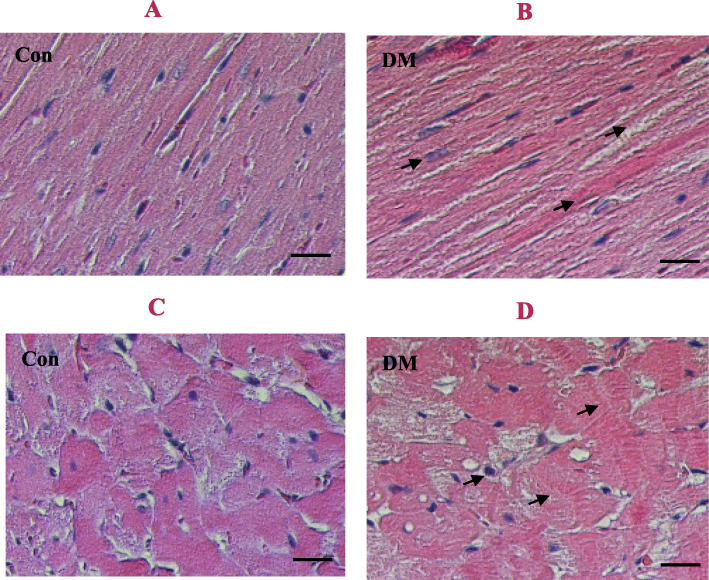
HE staining of myocardial tissue in T2DM rats. Panel **a** & **b** show the hematoxylin-eosin **(**HE**)** staining of left ventricular longitudinal sections in control and type 2 diabetic (T2DM) rats; Panel **c** & **d** represent the HE staining of left ventricular transverse sections in control and T2DM rats. Scale bar indicate 50 μm

As presented in Table 4, the ejection fraction (EF) and fraction shortening (FS) of left ventricle were notably reduced in T2DM rats compared with control rats (*P* < 0.01); the isovolumic relaxation time (IVRT) of left ventricle was significantly prolonged, while the E/A ratio of early (E) to late (A) left ventricular filling velocities was greatly decreased in diabetic rats compared with control rats (*P* < 0.01), indicating the impairments of left ventricular diastolic and systolic functions in diabetic rats. Not only end-systolic volume and end-diastolic volume (ESV & EDV) of left ventricle but also left ventricle internal diameters at end-systole and end-diastole (LVIDs & LVIDd) in diabetic rats were obviously larger than those of control rats (*P* < 0.01), indicating changes in left ventricular morphology in diabetic rats. However, there was no significant difference between the two groups either in left ventricle posterior wall thickness at end-systole and diastole (LVPWs & LVPWd) or interventricular septum thickness at end-systole and end-diastole (IVSs & IVSd, *P* > 0.05), suggesting that there is no obvious myocardial remodeling in the diabetic rats. Moreover, histological analysis by hematoxylin-eosin **(**HE**)** staining of cardiac sections showed a fewer myocardial cells, enlarged nucleus and widened the intercellular space in diabetic group compared with control group (Fig. 2), along with an increase of heart mass index (HMI), the ratio of heart weight (mg) to body weight (g) in diabetic rats compared with control rats (*P* < 0.01, Table 4). Taking together, these results suggest the cardiac hypertrophy.

**Table 4:** Cardiac dysfunction measured by echocardiography in T2DM rats

Parameters	Control	Diabetes
**HMI** (mg/g)	2.26 ± 0.09	2.84 ± 0.13**
**HR** (bpm)	379.31 ± 2.34	341.13 ± 22.43
**CO** (ml/min)	46.44 ± 3.29	43.74 ± 3.01
**EF** (%)	84.80 ± 2.58	54.05 ± 2.87**
**FS** (%)	55.19 ± 3.75	35.13 ± 0.76**
**IVRT** (ms)	22.67 ± 0.87	35.30 ± 1.37**
**E/A** (ratio)	1.66 ± 0.04	0.72 ± 0.04**
**ESV** (μl)	20.41 ± 4.16	105.86 ± 9.72**
**EDV** (μl)	140.34 ± 9.41	228.80 ± 9.54**
**LVIDs** (mm)	2.30 ± 0.18	4.37 ± 0.11**
**LVIDd** (mm)	5.16 ± 0.19	6.72 ± 0.16**
**LVPWs** (mm)	3.17 ± 0.13	3.38 ± 0.14
**LVPWd** (mm)	2.30 ± 0.13	2.32 ± 0.08
**IVSs** (mm)	2.99 ± 0.11	3.25 ± 0.07
**IVSd** (mm)	2.05 ± 0.06	2.22 ± 0.07

Data are expressed as mean ± *S.E.M.*, *n* = 5, **P < 0.01 vs Control

*T2DM* Type 2 diabetes mellitus, *HMI* Heart mass index, *HR* Heart rate, *CO* Cardiac output, *EF* Ejection fraction, *FS* Fractional shortening, *IVRT* Isovolumic relaxation time, *E/A* The ratio of early (E) to late (A) left ventricular filling velocities, *ESV or EDV* End-systolic or end-diastolic volume, *LVIDs or LVIDd* Left ventricle internal diameter at end-systole or end-diastole, *LVPWs or LVPWd* Left ventricle posterior wall thickness at end-systole or end-diastole, *IVSs or IVSd* Interventricular septum thickness at end-systole or end-diastole

### Suppression of cardiac mitochondrial biogenesis and function in T2DM rats

**Fig. 3:**
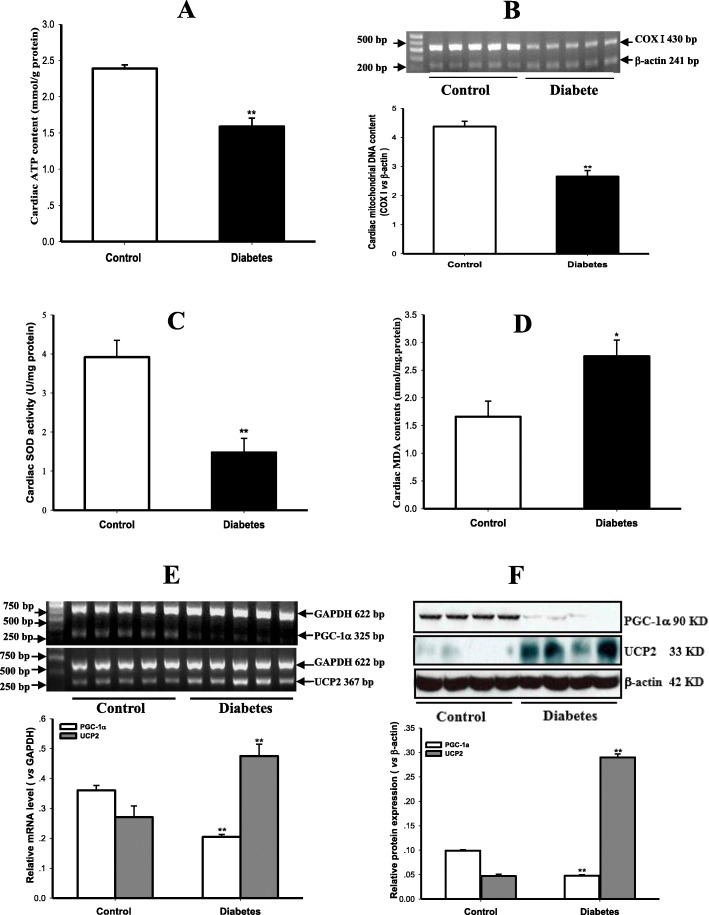
Impairments of mitochondrial function and biogenesis as well as their regulation in the myocardium of T2DM rats. Myocardial ATP content was measured to reflect mitochondrial function (panel **a**), and mitochondrial biogenesis was evaluated by mitochondrial DNA content, which was expressed by the copy number ratio of mitochondrial genes such as cytochrome C oxidase subunit I (COX I) and nuclear genes like β-actin indirectly (panel **b**), the activity of mitochondrial antioxidant enzyme manganese superoxide dismutase (Mn-SOD, panel **c**) and the content of lipid peroxidation product malondialdehyde (MDA, panel **d**) were determined to estimate oxidative stress, the transcription and protein expression of peroxisome proliferator-activated receptor-γ coactivator-1α (PGC-1α) & uncoupling protein 2 (UCP2) genes were detected by RT-PCR (panel **e**) and Western blotting (panel **f**) in the myocardium of control and type 2 diabetic (T2DM) rats. Graphs in panel ** b**, **e** & **f** represent the quantification of COX I vs β-actin, PGC-1α and UCP2 vs GAPDH (mRNA) or β-actin (Protein), respectively. Data are expressed as mean ± *S.M.E.*; *n* = 4 ~ 5. ^*^P < 0.05, ^**^P < 0.01 vs Control

In comparison with control rats, cardiac ATP content was remarkably reduced (Fig. 3a, *P* < 0.01), and the content of mtDNA, mirrored by the copy number ratio of COX I to β-actin, was extremely suppressed (Fig. 3b, *P* < 0.01) in the myocardium of diabetic rats, indicating the suppressions of myocardial mitochondrial function and biogenesis in T2DM rats. Myocardial Mn-SOD activity was decreased obviously (Fig. 3c, *P* < 0.01) while MDA content was increased (Fig. 3d, *P* < 0.05) in diabetic rats compared with control rats, showing an enhanced oxidative stress in T2DM rats. The transcription and expression of PGC-1α were apparently down-regulated, whereas UCP2 transcription and expression were evidently up-regulated in the myocardium of diabetic rats compared with control rats (Fig. 3e & f, all *P* < 0.01), suggesting an abnormal regulation of mitochondrial biogenesis and function in T2DM rats.

### Disorder of ADMA signal pathway in the myocardium of T2DM rats

**Fig. 4:**
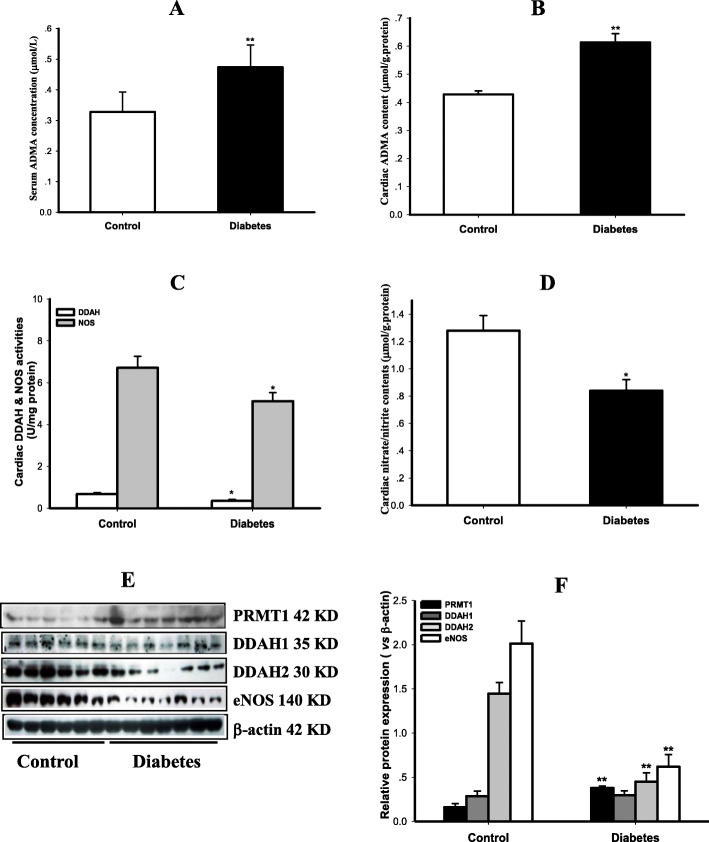
Disorder of ADMA signal pathway in the myocardium of T2DM rats. Levels of asymmetric dimethylarginine (ADMA) in serum and myocardium were measured in control and type 2 diabetic (T2DM) rats. Activities of dimethylarginine dimethylaminohydrolase (DDAH) and nitric oxide synthase (NOS) as well as content of nitric oxide (NO) reflected by its stable end products nitrite/nitrate were also detected; protein expressions of protein arginine N-methyltransferase 1 (PRMT1), DDAH 1 and DDAH2 as well as endothelial NOS (eNOS) were determined by Western blotting in the myocardium of control and T2DM rats. Panel A & B show the serum ADMA concentration and cardiac ADMA content; panel C displays the activities of DDAH and NOS, panel D presents nitrite/nitrate contents; panel E demonstrates the gel electrophoresis bands of indicated protein expression in the myocardium of rats, and panel F is the quantification of relative protein expression vs β-actin. Data are expressed as mean ± *S.M.E.*, *n* = 5 ~ 6. ^*^P < 0.05, ^**^P < 0.01 vs Control

As exhibited in Fig. 4, both serum ADMA levels and myocardial ADMA contents were significantly increased in T2DM rats compared with control rats (*P* < 0.01), indicating the accumulation of endogenous ADMA in diabetic rats. In contrast, not only activities of DDAH and NOS but also contents of NO metabolic products nitric/nitrate were decreased in the myocardium of T2DM rats compared to control rats (*P* < 0.05), suggesting the reduction of NO production in diabetic rats. In addition, Myocardial PRMT1 expression was up-regulated, whereas expressions of DDAH2 and eNOS, which are the principal isoforms of DDAH and NOS in cardiovascular system, were down-regulated in T2DM rats compared with control rats (*P* < 0.05). These results indicate that ADMA signal pathway was disordered in the myocardium of T2DM rats.

### The correlation of myocardial ADMA contents and cardiac dysfunction or mitochondrial dysfunction

Linear regression analysis suggested that myocardial ADMA content was negatively correlated not only with cardiac function indexes EF and ES, but also with contents of mtATP and mtDNA in control and diabetic rats. The Pearson’s correlation coefficients were − 0.905, − 0.873, − 0.820, − 0.839, respectively (*P* < 0.01). Furthermore, cardiac ATP and mtDNA contents were positively correlated with EF and ES, and the Pearson’s correlation coefficients were 0.839, 0.805, 0.909 and 0.871 (*P* ≤ 0.01). These results indicate that endogenous ADMA accumulation in myocardium of rats is closely related to not only cardiac dysfunction but also mitochondrial biosynthesis and dysfunction. Myocardial mitochondrial biogenesis and dysfunction are strongly associated with cardiac dysfunction.

### ADMA directly suppressed myocardial mitochondrial biogenesis in vitro and in vivo

**Fig. 5:**
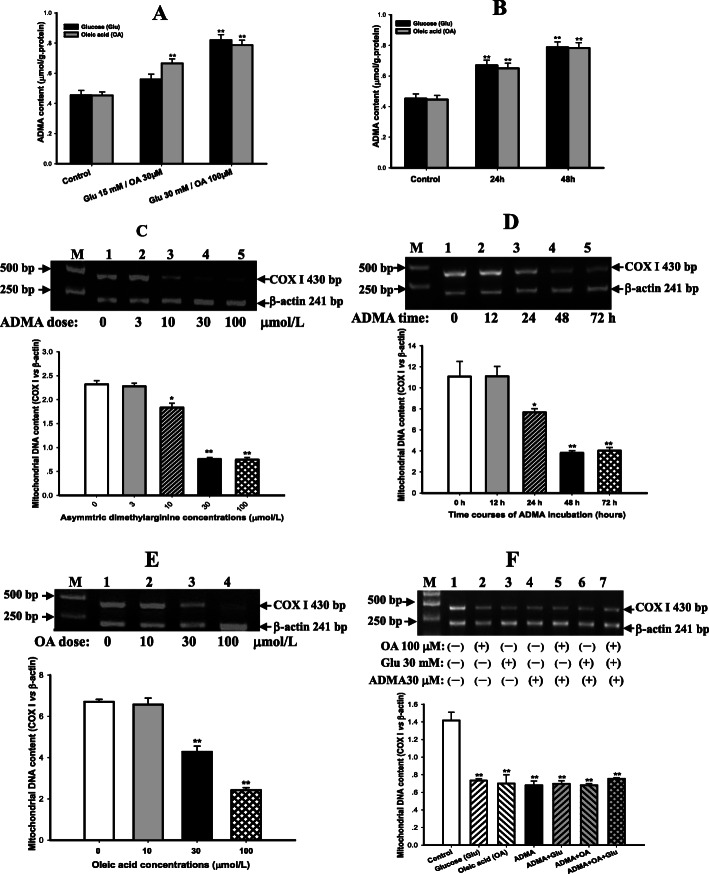
Dose- and time-dependent impairments of mitochondrial biogenesis in rat cardiomyocytes induced by ADMA and OA. Rat **c**ardiomyocytes were incubated with various dose of high glucose (15 ~ 30 mM) and asymmetric dimethylarginine (ADMA, 3 ~ 100 μM) or oleic acid (OA, 10 ~ 100 μM) and for different time (12 ~ 72 h) as indications. The content of ADMA was measured by enzyme linked immunosorbent assay (ELISA) and normalized to the protein content of the supernatants of cell lysis. The ratio of the **c**opy number of mitochondrial gene COX Ι to nuclear gene β-actin was used to evaluate the mitochondrial biogenesis. Panel **a** & **b** show the dose-response and time course of high glucose and OA on ADMA elaboration; Panel **c** & **d** represent the dose-response and time course of ADMA on mitochondrial biogenesis; Panel **e** displays the dose-response of OA, and panel **f** displays effects of ADMA combination with high glucose or OA or even both on mitochondrial biogenesis in cardiomyocytes. Graphs in panel **c**, **d**, **e** & **f** represent the quantifications of COX Ι vs β-actin from 3 independent experiments, respectively. Date are expressed as mean ± *S.E.M.*, *n* = 3, ^*^P < 0.05, ^**^P < 0.01 vs control or zero group

To observe the direct effect of ADMA on mitochondrial biogenesis **in vitro**, cardiomyocytes were incubated with various concentrations of exogenous ADMA (3 ~ 100 μM) or free fatty acid oleic acid (OA, 10 ~ 100 μM) for 48 h in dose-response experiment. Then 30 μM ADMA was chosen to treat cardiomyocytes for different time (12 ~ 72 h) in experiment of time course. Finally, cells were treated separately with 30 μM ADMA or 30 mM glucose or 100 μM OA and their various combinations for 48 h, respectively. As depicted in Fig. 5, incubation of cardiomyocytes with glucose and OA increased endogenous ADMA accumulation in a dose- and time-dependent manners (Fig. 5a & b, *P* < 0.01). Exogenous ADMA directly suppressed the mitochondrial DNA content in cultured cardiomyocytes in a dose- and time-dependent manners as well, especially at 30 μM ADMA for 48 h (Fig. 5c & d, *P* < 0.01). Accordingly, this dose and time were applied in the following experiments.

Treatment with OA (10 ~ 100 μM) for 48 h also inhibited mitochondrial DNA content in a dose-dependent manner. Treatment with 30 mM high glucose induced an equivalent inhibition on mitochondrial DNA content as 30 μM ADMA or 100 μM OA (Fig. 5e & f, *P* < 0.01). These results indicate that ADMA has similar inhibitory effect on mitochondrial biogenesis as high glucose or free fatty acid in cardiomyocytes. However, treatment with ADMA in combination with either high glucose or OA and even both of them no longer increased the inhibition of mitochondrial DNA content in cardiomyocytes (Fig. 5f), implying that ADMA may be a mediator of both high glucose and free fatty acid for inhibiting mitochondrial biosynthesis in rat cardiomyocytes.

**Fig. 6:**
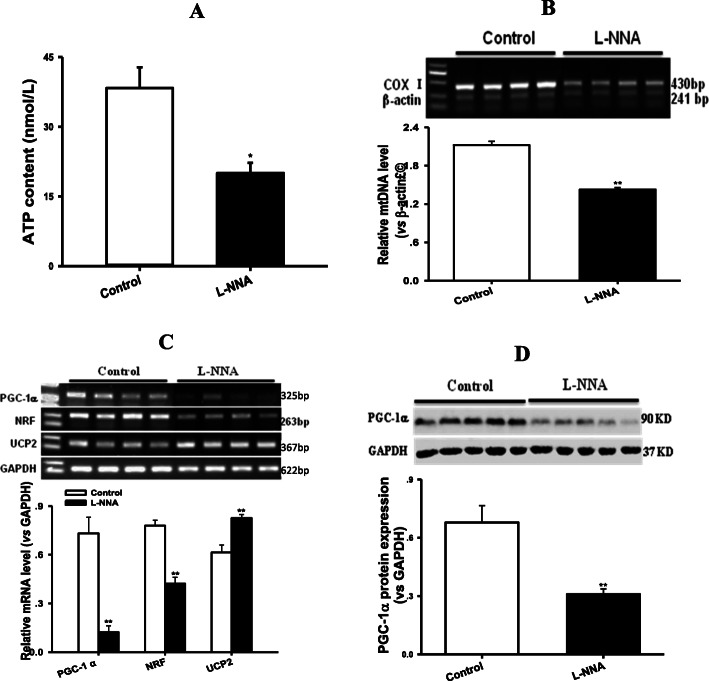
Exogenous NOS inhibitor L-NNA suppressed mitochondrial DNA and ATP contents, PGC-1α, NRF and UCP2 transcriptions, and PGC-1α expression in the myocardium of rats. Panel **a** shows ATP content, panel **b** represents mitochondrial DNA content, panel **c** exhibits the relative mRNA levels of PGC-1α, NRF and UCP2, panel **d** displays the relative protein level of PGC-1α in the myocardium of rats treated with L-Nitro-arginine (L-NNA, 50 mg/Kg/d, i.g.) for 8w, respectively. Graphs in panel **b** ~ **d** represent the quantifications of COX I vs β-actin (DNA), PGC-1α or NRF or UCP2 vs GAPDH (mRNA) and PGC-1α vs GAPDH (Protein), respectively. Data are expressed as mean ± *S.E.M.*, *n* = 4 ~ 5, ^**^P < 0.01 vs Control

To observe the direct effect of ADMA on mitochondrial biogenesis in vivo, normal rats were administrated by gavage of exogenous NOS inhibitor L-NNA (50 mg/kg/d) for 8w. As expected, administration of L-NNA also caused similar inhibitions of myocardial mitochondrial biosynthesis and function as well as their regulation disorders to T2DM rats (Fig. 6, *P* < 0.01). These results indicated that inhibition of NOS induced by either ADMA or L-NNA could suppress myocardial mitochondrial biogenesis in vitro or in vivo.

### ADMA suppressed the expression and transcription of PGC-1α via inhibiting its promoter activity while boosting its post-translational modification

**Fig. 7:**
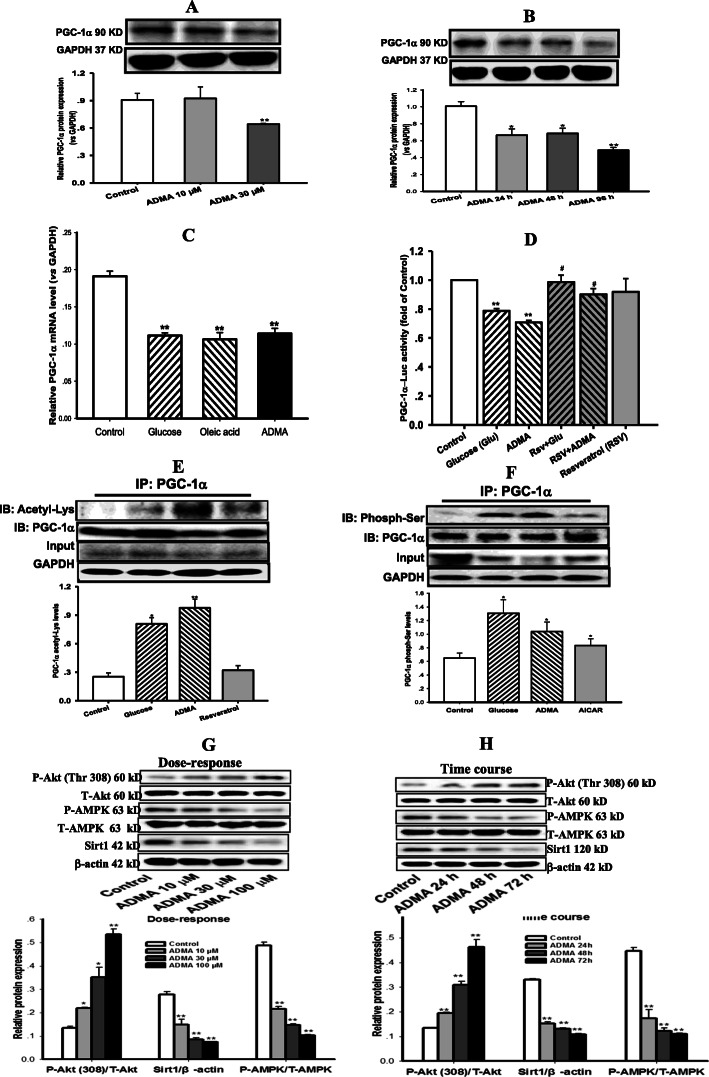
ADMA suppressed the expression and transcription of PGC-1α by inhibiting its promoter activity but boosting its protein acetylation and phosphorylation. Western blotting was applied to measure expressions of peroxisome proliferator activated receptor γ coactivator-1α (PGC-1α), Sirt1, total and phosphorylated Akt and AMPK proteins. Reversed transcription PCR was used to detect the transcription of PGC-1α, luciferase assay was performed to analyze the promoter activity of PGC-1α, and immunoprecipitation was employed to determine the post-translational modification including acetylation and phosphorylation of PGC-1α in cardiomyocytes incubated with ADMA (30 μM) and glucose (30 mM), or oleic acid (OA, 100 μM) for 48 h. Panel **a** & **b** demonstrate the dose-response (10 ~ 100 μM) and time course (24 ~ 72 h) of asymmetrical dimethylarginine (ADMA) on PGC-1α expression, respectively; Panel **c** shows the PGC-1α transcription, and panel **d** displays the promoter activity of PGC-1α. Panel **e** & **f** diagram the acetylation (resveratrol as the negative control) and phosphorylation (AICAR as the positive control) of PGC-1α protein, respectively. Panel **g** & **f** are the dose-response and time course of ADMA on expressions of sirt1, phosphorylated Akt and AMPK proteins. Graphs in panel **a** ~ **b**, **e** ~ **f** and **g** ~ **h** represent the quantification of PGC-1α vs GAPDH, acetylated and phosphorylated PGC-1α vs its total protein, phosphorylated Akt and AMPK vs their total proteins as well as sirt1 vs β-actin from 3 independent experiments, respectively. Date are expressed as mean ± *S.E.M.*, *n* = 3, ^*^P < 0.05, ^**^P < 0.01 vs control

As indicated in Fig. 7a & b, ADMA suppressed PGC-1α expression in a concentration- and time-dependent manners in cardiomyocytes (*P* < 0.01). PGC-1α transcription was also down-regulated in cardiomyocytes after exposure to ADMA or high glucose or OA for 48 h (Fig. 7c, *P* < 0.01). To further dissect the mechanism behind ADMA suppressing PGC-1α transcription and expression, the promoter activity of PGC-1α was detected by a dual-luciferase reporter assay. As expected, treatment of cardiomyocytes either with 30 μM ADMA or 30 mM glucose for 48 h remarkably reduced PGC-1α promoter activity (Fig. 7d, *P* < 0.01). Pretreatment of cardiomyocytes with resveratrol (RSV, 20 μM) for 2 h and co-incubation with ADMA or high glucose for 48 h could prevent the inhibition of PGC-1α promoter activity induced by ADMA or high glucose (Fig. 7d, *P* < 0.05). These results suggest that ADMA could inhibit PGC-1α promoter activity, leading to suppressions of PGC-1α transcription and expression in cardiomyocytes.

As disclosed in Fig. 7e & f, treatment of cardiomyocytes with ADMA or high glucose remarkably enhanced both acetylation and phosphorylation of PGC-1α protein (*P* < 0.05). Resveratrol, an agonist of deacetylase Sirt1 as the negative control of acetylation, did not increase PGC-1α acetylation (Fig. 7e, *P* > 0.05), whereas AICR, an agonist of AMPK as the positive control of phosphorylation, enhanced PGC-1α phosphorylation (Fig. 7f, *P* < 0.05) in cardiomyocytes. These results indicated that ADMA and high glucose could enhance PGC-1α acetylation and phosphorylation leading to suppressed coactivator activity in cardiomyocytes.

After incubation with ADMA, the ratio of phosphorylated Akt (P-Akt) / total Akt (T-Akt) expression was significantly increased, whereas the ratio of phosphorylated AMPK (P-AMPK) / total AMPK (T-AMPK) expression was notably suppressed in a dose- and time-dependent manners (Fig. 7g & h, *P*<0.01). Moreover, ADMA also significantly reduced Sirt1 expression in cardiomyocytes (Fig. 7g & h, *P* < 0.01). These results indicated that ADMA could down-regulate Sirt1 expression and up-regulate Akt activation resulting in enhances of PGC-1α acetylation and phosphorylation in cardiomyocytes.

### Rosiglitazone reversed ADMA-induced suppressions of mitochondrial biogenesis and function in cardiomyocytes

**Fig. 8:**
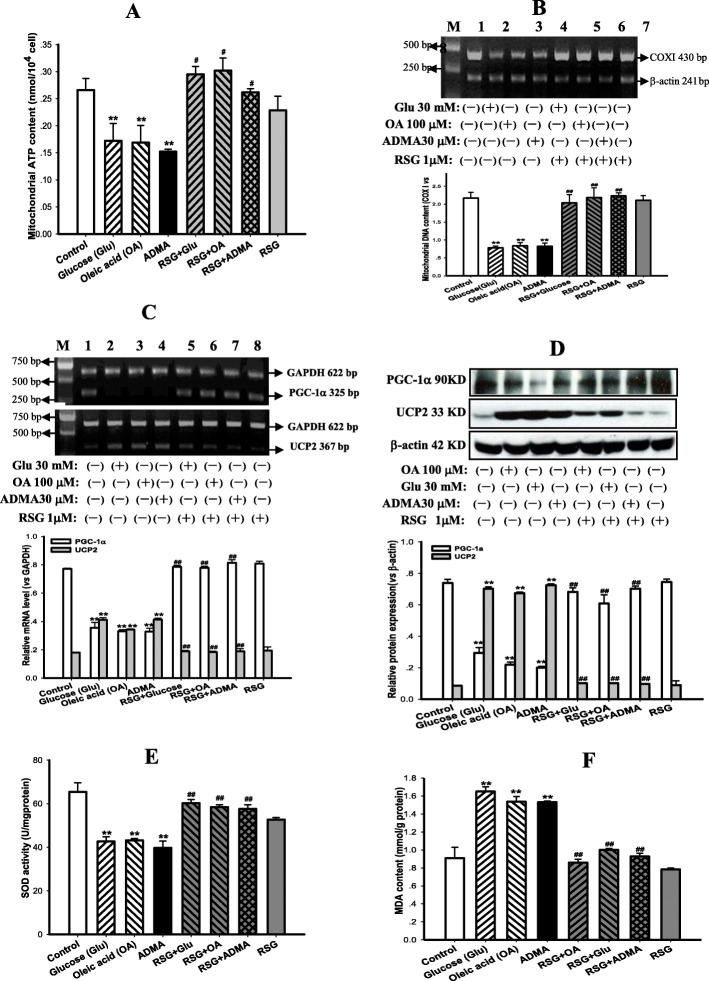
Rosiglitazone reversed inhibitions of mitochondrial function and biogenesis as well as their regulation disorders in cardiomyocytes incubated with ADMA. ATP content was measured to reflect mitochondrial function (Panel **a**), and the ratio of the DNA **c**opy number of COX Ι and β-actin genes was used to evaluate mitochondrial biogenesis (mtDNA, Panel **b**); Transcriptions (mRNA, Panel **c**) and expression (Protein, Panel **d**) of PGC-1α and UCP2 genes were detected to assess regulations of mitochondrial biogenesis and function; manganese superoxide dismutase (Mn-SOD) activity and malonyldialdehyde (MDA) content were measured to estimate oxidative stress (Pane **e** & **f**) in cardiomyocytes incubated without and with asymmetric dimethylarginine (ADMA, 30 μM) or high glucose (30 mM) or oleic acid (OA, 100 μM) for 48 h, and plus rosiglitazone (RSG, 1 μM) pretreatment for 2 h and then co-incubated with ADMA or high glucose or OA for 48 h, respectively. Graphs in panel **b**, **c** & **d** represent the quantifications of COX Ι vs β-actin (mtDNA), PGC-1α and UCP2 vs GAPDH (mRNA) or vs β-actin (Protein) from 3 independent experiments, respectively. Date are expressed as mean ± *S.E.M.*, *n* = 3, ^**^P < 0.01 vs control, ^*#*^*P* < 0.05, ^##^*P* < 0.01 vs glucose, OA or ADMA

As depicted in Fig. 8, contents of mtATP and mtDNA reflected by the copy number ratio of COX I to β-actin, were significantly suppressed in cardiomyocytes after incubation with ADMA (30 μM) or high glucose (30 mM) or high free fatty acid (100 μM OA) for 48 h compared with control group (Fig. 8a & b, *P* < 0.01). Furthermore, these incubations also down-regulated PGC-1α while up-regulated UCP2 transcription and expression in cardiomyocytes (Fig. 8c & d, *P* < 0.01). Treatment with rosiglitazone not only attenuated the suppressions of ADMA, high glucose and OA on mitochondrial function and biogenesis, but also recovered their disturbed regulation (Fig. 8a~d, *P* < 0.05 ~ 0.01). Moreover, oxidative stress was strongly enhanced as reflected by the lower Mn-SOD activity and the higher lipid peroxidation product MDA content in cardiomyocytes after being exposed to ADMA, or high glucose, or OA for 48 h compared with the control group (Fig. 8e & f, *P* < 0.01), indicating the increase of oxidative stress. Rosiglitazone treatment prevented the increased oxidative stress through greatly upping Mn-SOD activity and remarkedly downing MDA content (Fig. 8e & f, *P* < 0.01). However, rosiglitazone per se did not affect neither mitochondrial biogenesis and function nor their regulation and oxidative stress (Fig. 8a~f, *P* > 0.05). These results indicated that ADMA like high glucose or OA could directly suppress mitochondrial function and biogenesis as well as disturb their regulation in cardiomyocytes. These results also suggested that ADMA-induced inhibition of mitochondrial biogenesis might be via PGC-1α/PPARγ pathway. Rosiglitazone treatment could preserve mitochondrial biogenesis and function from the inhibition of ADMA or high glucose or OA, and normalize the disturbances of their regulation in cardiomyocytes.

## Discussion

It is well known that diabetic patients are susceptible to DCM. The present study displayed a significant decrease in cardiac diastolic and systolic functions in T2DM rats compared with control rats. These results are consistent with the cardiac dysfunction data previously reported in various T2DM animal models and even in T2DM patients [26, 29, 30], indicating the formation of DCM in T2DM rats of this study. Although the pathogenesis of DCM is not completely understood, emerging evidence demonstrate that mitochondrial dysfunction plays an essential role in the development of DCM [2, 3]. Zhang X et al recently reported that treatment of diabetic rabbits with dipeptidyl peptidase-4 inhibitor could prevent cardiac dysfunction, either preserve myocardial mitochondrial function or improve mitochondrial biosynthesis [31]. The present study demonstrated the suppressed mitochondrial biogenesis, impaired mitochondrial function, and increased oxidative stress in the myocardium of T2DM rats. Linear regression analysis showed that cardiac mtATP and mtDNA contents were positively correlated with cardiac functions, suggesting that impairments of myocardial mitochondrial biogenesis and function contributed to the cardiac dysfunction in T2DM rats. Similar impairments of mitochondrial biogenesis and function assessed by different parameters were previously reported in the myocardium, liver and skeletal muscle of T2DM rats or mice [3, 21, 29]. In addition to the decrease in PGC-1α expression in the heart of diabetic mice or increase in UCP2 expression in the heart of diabetic rats previously reported [3, 29], the present study also showed both down-regulation of PGC-1α expression and up-regulation of UCP2 expression in the myocardium of T2DM rats. These results indicated that the regulations of mitochondrial biogenesis and function had been disrupted in the myocardium of T2DM rats. However, the further mechanisms leading to these impairments are not known yet. The present study demonstrated for the first time that the accumulation of endogenous ADMA in the myocardium of T2DM rats was not only closely related to the suppressions of mitochondrial biogenesis and function, but also associated with cardiac dysfunctions. Therefore, we propose that ADMA may be a key player in the impaired mitochondrial biosynthesis and function of DCM.

To determine the causal relationship between ADMA and impairments of mitochondrial biosynthesis, the present study investigated the direct effects of both endogenous NOS inhibitor ADMA and exogenous NOS inhibitor L-NNA, a homolog of ADMA, on mitochondrial biosynthesis and its regulation in vitro and in vivo. Results revealed that ADMA could directly suppress mtDNA content along with PGC-1α transcription and expression in a dose- and time-dependent manners in cardiomyocytes. Administration of L-NNA to normal rats could also cause similar inhibitions of myocardial mitochondrial biosynthesis and function as well as PGC-1α transcription and expression to T2DM rats. These results indicate that the accumulation of endogenous NOS inhibitor ADMA is the fundamental cause of impaired mitochondrial biogenesis in the myocardium of T2DM rats. Interestingly, high glucose and free fatty acid had the similar inhibitory effects on mitochondrial biogenesis as ADMA, but treatment with ADMA in combination with high glucose or OA and even both of them, no longer increased the inhibition of mitochondrial biosynthesis, implying that ADMA may be a mediator of high glucose and free fatty acid-induced inhibition of mitochondrial biosynthesis in cardiomyocytes. Indeed, incubation of cardiomyocytes with various dose of high glucose (15 ~ 30 mM) and oleic acid (30 ~ 100 μM) for 24 ~ 48 h could increase endogenous ADMA elaboration in a dose- and time-dependent manners. Similar results had been reported previously in cultured endothelial cells after exposure to high glucose [32, 33]. Owing to ADMA-induced reduction of PGC-1α transcription, the effect of ADMA on the promoter activity of PGC-1α was detected in this study to dissect the further mechanism for ADMA suppressing PGC-1α transcription and expression. As expected, both ADMA and high glucose could noticeably inhibit the promoter activity of PGC-1α, and this inhibition was attenuated by the pretreatment with Sirt1 agonist resveratrol. Previous study reported that Sirt1 overexpression and resveratrol treatment remarkably increased PGC-1α promoter activity in cultured mouse myoblastic C2C12 cells while transcription and expression of PGC-1α were significantly reduced in skeletal muscle of SIRT1 homozygous-null mice [34]. Hence ADMA-induced inhibition of PGC-1α promoter activity could be considered as one of reasons for suppressions of PGC-1α transcription and expression in myocardium of T2DM rats.

It has been documented that the transcriptional activity of PGC-1α could be decreased by its protein acetylation, while Sirt1-catalyzed deacetylation leaded to an increase in its transcriptional activity [8]. AMPK-induced PGC-1α phosphorylation leaded to the enhance, whereas Akt-induced phosphorylation resulted in the decrease of its transcriptional activity [9, 10]. The current study discovered that ADMA and high glucose remarkably enhanced PGC-1α acetylation but resveratrol did not. In addition, ADMA also inhibited Sirt1 expression in cardiomyocytes in a dose- and time-dependent manners. These results indicated that ADMA promotes PGC-1α acetylation, which might be caused, at least in part by the inhibition of Sirt1 expression, despite the fact that expression of acetylase was not detected in this study. Furthermore, both ADMA and high glucose also augmented PGC-1α phosphorylation. This augmentation was similar to that of AMPK activator AICAR, but ADMA did not activate AMPK other than upregulated Akt phosphorylation at the site of 308 threonine in a dose- and time-dependent manners. These results suggested that ADMA promotes PGC-1α phosphorylation not by activating AMPK but by Akt activation, which may result in the inhibition of PGC-1α transcriptional activity [10]. Taking together, ADMA boosting PGC-1α acetylation and phosphorylation could be considered as another reason for suppressing PGC-1α transcription and expression in myocardium of T2DM rats.

Rosiglitazone is an effective anti-diabetic drug acted on increasing insulin sensitivity. Rong et al previously reported that rosiglitazone as an agonist of PPARγ could improve impairments of mitochondrial biogenesis and PGC-1α expression in the adipose tissue of T2DM mice [23]. Zhang & Wang et al also demonstrated that treatment with rosiglitazone could protect vascular endothelial function in rats fed with HFD and patients with metabolic syndrome [35, 36]. Therefore, the present study was to determine whether rosiglitazone could revise the effect of ADMA in rat cardiomyocytes. It was found that rosiglitazone treatment not only amended ADAM- or high glucose-induced impairments of mitochondrial function and biogenesis but also corrected their regulation disorders in ADMA-incubated cardiomyocytes. These results suggested not only ADMA-induced inhibition of mitochondrial biogenesis via PGC-1α/PPARγ pathway in cardiomyocytes but also the potential application of rosiglitazone or other thiazolidinediones in DCM control.

## Conclusion

In summary, this study demonstrated for the first time that the accumulation of endogenous NOS inhibitor ADMA in the myocardium of T2DM rats was not only closely related to suppressions of mitochondrial biogenesis and function, but also strongly associated with cardiac dysfunctions. Exogenous ADMA or L-NNA could directly inhibit mitochondrial biosynthesis and ATP production in cardiomyocytes or in rats. These effects of ADMA in vivo and in vitro were associated with either up-regulation of UCP2 transcription and expression or down-regulation of PGC-1α transcription and expression, which might be caused by the inhibition of PGC-1α promoter activity and boosting of PGC-1α acetylation and phosphorylation in cardiomyocytes. Pretreatment of cardiomyocytes with the PPARγ agonist rosiglitazone could revise ADMA-induced suppressions of mitochondrial biogenesis and function. These findings indicate an important role of ADMA in development of DCM and the potential for diagnosis and treatment new approaches of DCM by targeting ADMA and/or its signal pathway.

## Data Availability

All data generated or analyzed during this study are included in this published paper or are available from the corresponding author on reasonable request.

## Abbreviations

ADMA: Asymmetric dimethylarginine
Akt: Pprotein kinase B
AMPK: Adenosine monophosphate-activated protein kinase
AUC: Area under the curve
COX I: Cytochrome C oxidase subunit I
DCM: Diabetic cardiomyopathy
DDAH 1&2: Dimethylarginine dimethylaminohydrolase 1&2
EF: Ejection fraction
FBG: Fasting blood glucose
FIns: Fasting plasma insulin
FS: Fraction shortening
HDL: High density lipoprotein
HFD: High fat diet
LDL: Low density lipoprotein
MDA: Malondialdehyde
Mn-SOD: Manganese-superoxide dismutase
mtATP: Mitochondrial ATP
mtDNA: Mitochondrial DNA
NO: Nitric oxide
NOS: Nitric oxide synthase
OA: Oleic acid
OGTT: Oral glucose tolerance test
PGC-1α: Peroxisome proliferator-activated receptor-γ coactivator-1α
PRMT1: Protein arginine N-methyltransferase 1
ROS: Reactive oxygen species
Sirt1: Silent information regulator 2 homolog 1
T2DM: Type 2 diabetes mellitus
TC: Total cholesterol
TG: Triglycerid
UCP2: Uncoupling protein 2
